# Pulmonary tuberculosis and melioidosis coinfection in Brunei Darussalam: the importance of awareness and screening

**DOI:** 10.5365/wpsar.2022.13.4.957

**Published:** 2022-12-19

**Authors:** Abdur Rahman Rubel, Babu Ivan Mani, Panduru Venkata Kishore, Vui Heng Chong

**Affiliations:** a Department of Medicine, Pengiran Muda Mahkota Pengiran Muda Haji Al-Muhtadee Billah Hospital, Tutong, Brunei Darussalam.; b Department of Medicine, Raja Isteri Pengiran Anak Saleha Hospital, Bandar Seri Begawan, Brunei Darussalam.

## Abstract

Both tuberculosis (TB) and melioidosis are endemic to certain parts of the world, including Brunei Darussalam, with TB being more widespread. Despite this, coinfection with TB and melioidosis is rarely encountered and reported. Although still uncommon, there has been an increase in the number of cases of this coinfection reported during the past 10 years, all of which have been in India and the World Health Organization’s Western Pacific Region. We report a case of coinfection with pulmonary TB and melioidosis in a patient with poorly controlled diabetes mellitus. This 64-year-old man presented with symptoms and radiological features of pulmonary TB, confirmed by sputum smear, but sputum culture also yielded *Burkholderia pseudomallei*, the pathogen that causes melioidosis. Coinfection was detected due to our practice of routinely screening for other infections in patients suspected or confirmed to have pulmonary TB. This highlights the importance of awareness of melioidosis and the need to consider screening for infection, especially in endemic regions.

Tuberculosis (TB) is endemic to many underdeveloped and developing nations, while melioidosis is endemic only to certain tropical regions, in particular Thailand, northern Australia and south-eastern Asia. (*1*, *2*) Of these two infections, TB is by far more common and is encountered almost everywhere in the world. Melioidosis is less common but is now being reported in traditionally non-endemic countries due to increasing population movement. (*3*) Melioidosis is caused by *Burkholderia pseudomallei*, a Gram-negative, aerobic, saprophytic, non-fermenting bacillus commonly found in the soil in endemic regions, especially in irrigated agricultural fields and in surface water. The incidence of melioidosis increases during the wet season. (*2*) It is usually transmitted by inhalation; direct contact with infected rodents, food, soil, water or excreta; and inoculation from contaminated soil through abrasions or lesions in the skin. (*2*) The most common risk factors are occupational exposure, alcoholism and immunosuppressive conditions, such as diabetes mellitus (DM), renal disease and thalassaemia. (*2*) In Brunei Darussalam, the most commonly associated risk factors are DM, end-stage renal disease and thalassaemia. (*4*)

Melioidosis has a wide range of manifestations, including asymptomatic infection, but the most common is chronic pneumonia that mimics TB, as well as localized skin ulcers or abscesses, and fulminant septic shock with multiple abscesses in internal organs. Melioidosis has been reported to manifest up to decades after initial exposure. (*2*) TB and melioidosis share many similarities, from risk factors to varied manifestations that can pose a diagnostic challenge, especially in non-endemic countries where there is less awareness of melioidosis. Despite both TB and melioidosis being endemic in some countries, including Brunei Darussalam and other parts of the World Health Organization’s Western Pacific Region, coinfection is uncommon. To date, there have been only 13 cases of coinfection reported in the literature, with most reported during the past 10 years, highlighting a recent increase and the importance of awareness of these coinfections. (*5*-*14*) This report documents a case of coinfection with pulmonary TB (PTB) and melioidosis in a patient with poorly controlled DM in Brunei Darussalam.

## CASE REPORT

A 64-year-old man with type II DM, hypertension and dyslipidaemia was admitted with a chronic cough of  3 months’ duration that had recently worsened, becoming more productive with sputum. Sputum had been purulent and mixed with fresh blood for 1 week. He also had moderate weight loss of around 5 kg in 3 months. He had been an ex-smoker for more than 10 years and had no history of alcohol consumption. He had a long-standing history of poorly controlled DM (defined as glycated haemoglobin, or HbA1c, > 8.5%) and was noncompliant with his medications. He had retired from office work 6 years previously and had never done any farming or agricultural work. He had no history of TB or contact with patients with PTB.

**Fig. 1:**
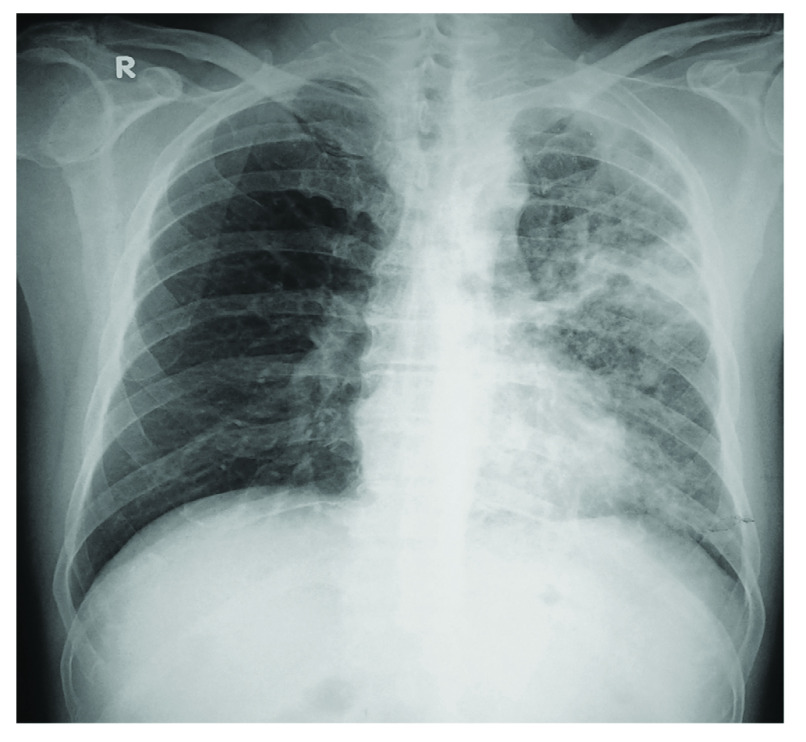
Initial chest X-ray of a 64-year-old man showing extensive changes in the left lung field, including small cavities

On examination, he was afebrile (temperature,35.6 °C) and was not in any distress. He had mild pallor but no cyanosis, clubbing or lymphadenopathy. There were coarse crepitations and mild rhonchi heard over the left lung fields. Laboratory investigation showed normal haemoglobin (13.1 g/dL, normal range [NR]: 13.5–17.9), liver function (including serum albumin) and renal function tests, total leukocyte count (67.4% neutrophils, 11.6% monocytes, 17.9% lymphocytes, 2.3% eosinophils), and an elevated erythrocyte sedimentation rate (66 mm/hour, NR: 1–20) and blood glucose (21.2 mmol/L, NR: 4–7.7). Serum HbA1c was very high at 15% (NR: 6.5%, preferred < 7.2% for DM). Chest X-ray (CXR) showed extensive interstitial opacities as well as cavities (**Fig. 1**). Ultrasonography of the abdomen and pelvis was normal.

[**insert** Figure 1]

**Fig. 2:**
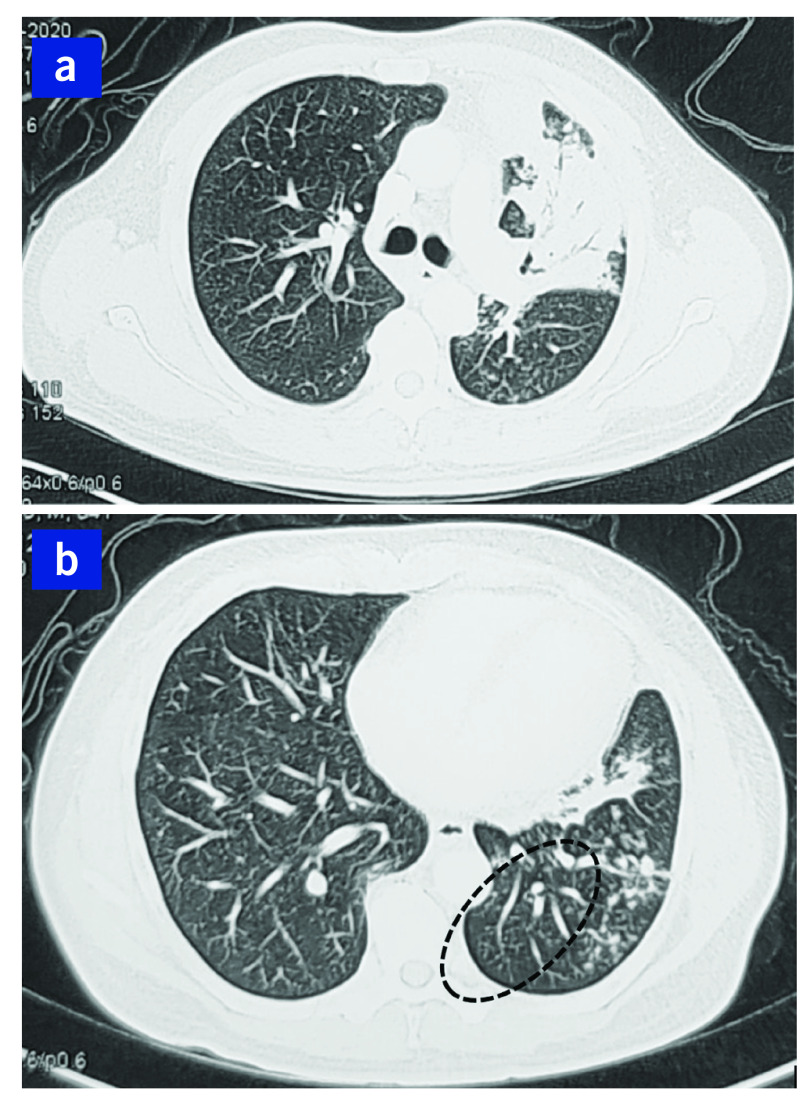
Axial computed tomography of a 64-yearold man showing (a) consolidation of the left upper lobe with volume loss and (b) the pulmonary tree-in-bud sign associated with tuberculosis affecting the peripheral pulmonary parenchyma (dotted oval)

PTB was suspected, and three consecutive morning sputum samples were sent for acid-fast bacilli (AFB) smear and culture, as well as bacterial culture, as part of our routine protocol. All three specimens were positive for AFB on Ziehl–Neelsen staining. A line-probe assay (GenoType MTBDRplus version 2, Hain Lifescience, Nehren, Germany) did not detect resistance to isoniazid or rifampicin. In addition to *Mycobacterium tuberculosis*, sputum microbial culture also isolated *B. pseudomallei*. Identification was done with standard techniques using Gram staining and biochemical reactions (i.e. auramine stain and antibiotic susceptibility testing). Antibiotic susceptibility testing was performed and interpreted using VITEK 2 and API 20NE (bioMérieux, Marcy-l’Étoile, France), and testing showed universal resistance to aminoglycosides. Blood culture was negative for *B. pseudomallei*. Sputum culture for mycobacteria confirmed pansensitive *M. tuberculosis*. Screening tests for HIV and hepatitis B and C were negative. Computed tomography (CT) imaging of the thorax, abdomen and pelvis, to assess for melioidosis involvement in other organs, showed only consolidation with volume loss in the left upper pulmonary lobe and the tree-in-bud sign (**Fig. 2**). There was no intra-abdominal organ involvement.

[**insert** Figure 2]

**Fig. 3:**
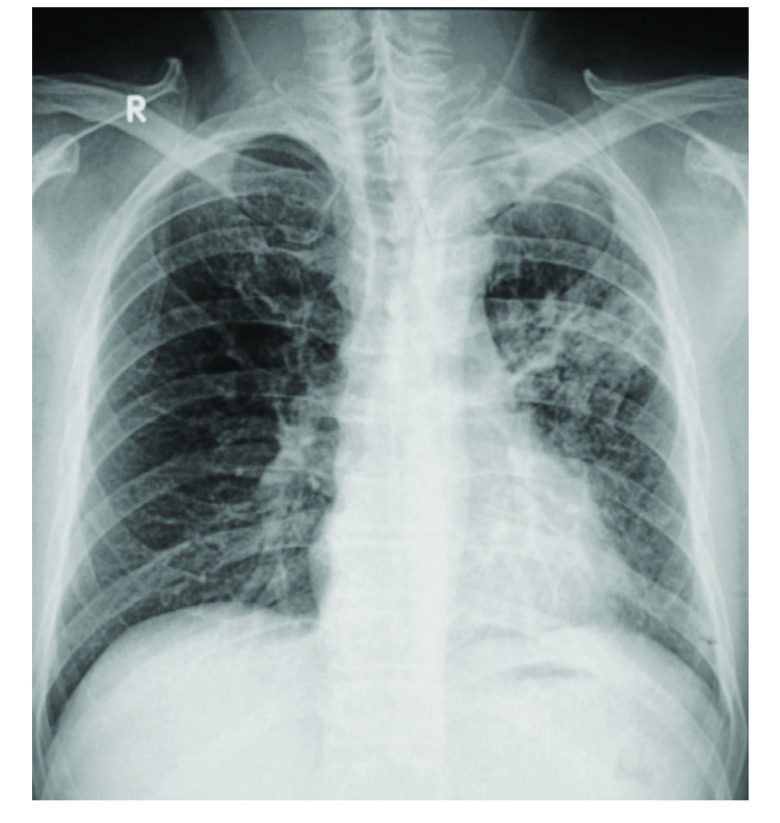
Repeat chest X-ray of a 64-year-old man done almost 3 months after the first X-ray, showing improvement

The patient was treated for coinfection with melioidosis and PTB. He was started on an intensive phase of intravenous ceftazidime (2 mg three times daily for 3 weeks), followed by maintenance therapy with a double dose of co-trimoxazole (sulfamethoxazole + trimethoprim, 800/160 mg twice daily for 10 weeks). He was also started on standard anti-TB therapy: an intensive phase with rifampicin (600 mg daily), isoniazid (300 mg daily), pyrazinamide (1.5 g daily) and ethambutol (900 mg daily) for 3 months and streptomycin (1 g daily) for 1 month, followed by a continuation phase with rifampicin (600 mg daily), isoniazid (300 mg daily) and ethambutol (900 mg daily) for 6 months. Pyridoxine 25 mg once daily was also given. Weekly liver profile monitoring for 1 month showed no hepatotoxic effects. While the patient was hospitalized, hyperglycaemia was managed with dietary adjustments and insulin therapy, and he was later converted to using oral hypoglycaemic agents. He became symptom-free within 7 days of starting treatment. He was discharged after three consecutive negative sputum AFB tests, and he continued the stipulated period of directly observed therapy for PTB and melioidosis treatment. A repeat CXR 3 months after the first showed marked improvement (**Fig. 3**). He completed 9 months of treatment for PTB and 3 months of melioidosis treatment, and was completely recovered at the end of therapy.

[**insert** Figure 3]

## Discussion

Both PTB and melioidosis are common in Brunei Darussalam. (*4*, *15*) TB is a notifiable disease in Brunei Darussalam, whereas melioidosis is not. Annually, an average of 227 TB cases were recorded in Brunei Darussalam between 2013 and 2018, for a rate of 54/100 000 population per year. (*15*) The incidence of melioidosis has been increasing in Brunei Darussalam from 2.9/100 000 in 1993 to 16.3/100 000 in 2018. (*4*) Our case represents the second case of coinfection with TB and melioidosis reported in Brunei Darussalam. The first case, reported in 2020, was in a 39-year-old woman with poorly controlled DM and manifested in a neck abscess. (*13*) In that case, melioidosis was initially diagnosed and only after surgical drainage and histological examination of the resected tissue was TB diagnosed. As in other countries, poorly controlled DM is the most important risk factor for melioidosis in Brunei Darussalam. (*4*) Similarly, DM is an important risk factor for TB, and one third of our patients with TB already have DM or are diagnosed within 6 months of their TB diagnosis. (*15*)

A literature search identified 10 reports of 13 cases of coinfection with TB and melioidosis (**Table 1**). (*5*-*14*) All but one occurred in males; their ages ranged from 39 to 64 years, and DM was the most common risk factor. Not all patients reported environmental or occupational exposure. Pulmonary coinfection was the most common manifestation, followed by neck abscess. All patients were successfully treated. The patient in the case reported by Kim et al. (*10*) was initially treated for PTB, but symptoms and pulmonary changes persisted. Pulmonary melioidosis was diagnosed only after surgical resection. In that case, the interval between the diagnosis of PTB and of melioidosis was 1 year. Whether pulmonary melioidosis was already present at the time of PTB diagnosis is unknown; the patient’s only risk factors were working as a welder and travelling to the Philippines, another country endemic for both TB and melioidosis.

**Table 1:** Summary of 14 reported cases of coinfection with tuberculosis and melioidosis

Author	Year	Country	No. of cases	Age/sex	Risk factors	Occupation	Manifestation		Outcome
							TB	Melioidosis	
Azali et al. (*5*)	2007	Malaysia	1	49/M	NA	NA	Hepatic (histology showing acid-fast bacilli)	Liver abscess (pus)	Survived
Shenoy et al. (*6*)	2009	India	1	40/M	Diabetes	Agriculture (paddy field)	Neck abscess	Neck abscess	Survived
Shetty et al. (*7*)	2010	India	1	40/M	Diabetes	Agriculture	Pulmonary (diagnosed by bronchoalveolar lavage)	Pulmonary (sputum)	Survived
Sulaiman et al. (*8*)	2013	Malaysia	1	54/M	Diabetes	Palm oil plantation worker	Cervical abscess	Cervical abscess	Survived
Sankar et al. (*9*)	2014	India	3	NA	NA	NA	NA	NA	NA
Kim et al. (*10*)	2015	Republic of Korea	1	60/M	None	Welder	Pulmonary	Pulmonary	Survived
Patra et al. (*11*)	2017	India	2	NA	NA	NA	Pulmonary	Pulmonary	NA
San Martin et al. (*12*)	2018	Philippines	1	59/M	None	NA	Pulmonary	Pulmonary and cutaneous (soft tissue)	Survived
Yap et al. (*13*)	2020	Brunei Darussalam	1	39/F	Diabetes (newly diagnosed)	Clerk in agriculture department	Neck	Neck	Survived
Tan (*14*)	2020	Singapore	1	64/M	Alcohol	Cable joiner	Disseminated: gastrointestinal, pulmonary, splenic	Splenic	Survived
Current report	2022	Brunei Darussalam	1	64/M	Diabetes	Retired office worker	Pulmonary	Pulmonary	Survived

F: female; M: male; NA: not available; TB: tuberculosis.

One study from India reported on melioidosis during an 8-year period (2007–2015): only two cases of coinfection occurred in 65 cases of pulmonary melioidosis, for a prevalence of 3.1%. (*11*) Another study from India looked at 301 patients with pyrexia of unknown origin and identified three cases of coinfection, for a prevalence of 1%. (*9*) A mathematical modelling study from Thailand, where TB and melioidosis are endemic, estimated a very low coinfection rate of 0.0085/100 000 population compared with 4.96/100 000 for melioidosis and 171/100 000 for TB. (*16*)

As with any infectious disease, awareness is important. Although melioidosis is increasingly reported in non-endemic regions, awareness is mainly limited to endemic regions. (*2*) Despite the availability of treatment, the fulminant form of melioidosis is associated with 40% mortality. (*2*) In contrast, TB is well recognized and awareness is almost universal. (*1*) Similar to melioidosis, manifestations of TB are varied and any organ can be affected. TB is still associated with significant morbidity and remains an important cause of mortality, especially if left untreated or if treatment is delayed.

Diagnosing PTB is often straightforward, but extrapulmonary TB can be extremely difficult, especially if laboratory confirmation is needed because laboratory investigations may overlap for the two types of TB. Chest imaging such as CXR can aid in diagnosis because for PTB it may show upper lobe changes and manifestations such as fibrosis and bronchiectasis. CT imaging can also provide useful clues: active PTB is often associated with the tree-in-bud sign, (*17*) as seen in our patient. However, this sign can also be seen in other pulmonary pathologies. Other changes include lobular consolidation, cavitation and bronchial wall thickening. (*17*) Melioidosis, however, is not associated with any typical chest manifestations, and manifestations can range from minimal changes to consolidation of the lung resembling PTB as well as community-acquired pneumonia. (*18*) The involvement of multiple sites, including the lung, liver and spleen, is common; for example, the honeycomb sign in the liver is typical of melioidosis (*18*) and can be detected by CT imaging. In cases of coinfection, the typical features of each infection may not be present, and often laboratory confirmation is required.

Given the increasing prevalence of DM, it is likely that TB, melioidosis and coinfection with both may also increase, as is evident by the rising number of reported cases. (*4*-*13*) Apart from the cases of coinfection documented in India, the remainder were from the Western Pacific Region. Therefore, in endemic countries, it is important to screen for coinfection with TB and melioidosis in patients with risk factors. In our setting, patients diagnosed with TB are routinely screened for DM or their level of control of DM is assessed. However, we do not routinely screen patients with DM for PTB unless they have symptoms or findings that are suggestive of TB. In non-endemic countries, awareness of melioidosis is important, and screening should be considered, especially for patients with relevant risk factors, such as DM or a history of travel to melioidosis-endemic countries. This is particularly important because both infections are treatable.

In conclusion, coinfection with TB and melioidosis is rarely encountered despite some countries being endemic for both. Most of the reported cases have occurred during the past decade, mostly in India and increasingly in the Western Pacific Region. Coinfection occurs more often in males, and DM is the most common risk factor. TB and melioidosis are treatable but require early diagnosis. To date, the outcomes of patients with this coinfection have been favourable, but these results are based on a small number of cases. As the number of reported cases increases, awareness of melioidosis becomes more important, and screening for coinfection should be considered, especially in patients with risk factors. Further studies exploring the outcomes of patients with coinfection will be needed.
